# Content validity of the Migraine-Specific Quality of Life Questionnaire version 2.1 electronic patient-reported outcome

**DOI:** 10.1186/s41687-019-0138-x

**Published:** 2019-07-11

**Authors:** Rebecca M. Speck, Huda Shalhoub, David W. Ayer, Janet H. Ford, Kathleen W. Wyrwich, Elizabeth N. Bush

**Affiliations:** 1 Evidera, 7101 Wisconsin Avenue, Suite 1400, Bethesda, MD 20814 USA; 2 Eli Lilly & Company, 893 S. Delaware Street, Indianapolis, IN 46225 USA

**Keywords:** MSQ, Content validity, ePRO, Usability

## Abstract

**Purpose:**

A concept elicitation, cognitive debriefing, and usability study was undertaken to: 1) ascertain the migraine experience with a particular focus on the impact on roles and daily functioning; 2) determine the comprehensiveness and comprehensibility of the Migraine-Specific Quality of Life Questionnaire version 2.1 electronic patient-reported outcome Role Function-Restrictive (MSQ v2.1 ePRO RFR) domain items, and the appropriateness and understanding of the recall period, response options, and instructions; and 3) assess the usability on an electronic tablet device.

**Methods:**

Eleven US English-speaking people with episodic or chronic migraine were recruited to participate in one-on-one interviews, encompassing methods appropriate for concept elicitation, cognitive debriefing, and usability testing. Interviews were audio-recorded, transcribed, and analyzed following the constant comparative method.

**Results:**

Participants (seven episodic and four chronic) had a mean age of 34.8 years, and nine were female. Through spontaneous mention or probing, the concepts of the MSQ v2.1 ePRO RFR domain items were described and endorsed by all participants as day-to-day functioning restrictions; except for item 5 (ability to concentrate), which was endorsed by 10 of 11 participants. Cognitive interviewing confirmed the MSQ v2.1 ePRO instructions were clear, meaningful, and important to assess as daily functioning impacts experienced as a result of migraine. Overall impressions of the ePRO device were favorable, and no participants reported any difficulties with use.

**Conclusions:**

The MSQ v2.1 ePRO RFR domain is content-valid and appropriate for inclusion in future studies designed to measure the functional impact of episodic or chronic migraine on the performance of day-to-day activities.

## Introduction

Migraine attacks are commonly described as having wide-ranging impacts, with adverse effects on physical and social aspects of daily living, including family, work, and social relationships; notably, even moderate migraine attacks can disturb patients’ normal activities [1]. The pain and associated symptoms during migraine attacks can be disabling, impairing patients’ ability to function [2]. In addition, patients can have significant impairment between attacks (interictal burden), including anticipation of the next attack, avoidance of activities due to fear of a migraine attack, and feeling drained from the experience [3–6]. Averting migraine-related disability is one of the primary treatment goals per migraine preventive treatment guidelines; yet, the current level of impaired functioning and disability among the migraine population remains high, indicating a continuing unmet medical need for these patients [7, 8].

Three phase 3 studies were designed to investigate the superiority of galcanezumab (120 mg/month or 240 mg/month) to placebo in the prevention of migraine among patients with episodic or chronic migraine. A key secondary endpoint of the three trials was an electronic patient-reported outcome (ePRO) instrument, the Migraine-Specific Quality of Life Questionnaire version 2.1 (MSQ v2.1) Role Function-Restrictive (RFR) domain, which measures the impact of migraine on work or daily activities, relationships with family and friends, leisure time, productivity, concentration, energy, and tiredness. The original MSQ v1.0, developed in 1992 by Glaxo Wellcome Inc., was a 16-item instrument consisting of three domains. The MSQ v1.0 was developed based on a combination of a literature review and discussions with migraine specialists and patients [9]. The final items selected were based on 25 one-on-one patient interviews, wherein interviewees were asked to comment on content, difficulty, comprehensiveness, and appropriateness of proposed response categories. This work informed the development of the instrument and confirmed the appropriateness of its items, recall period, and response options. Additional development research was conducted in the mid-1990s on the MSQ v1.0, incorporating feedback from clinicians and patients enrolled in five clinical trials and an additional 30 one-on-one patient interviews, resulting in revisions to the MSQ (v2.0) [10]. Subsequently, the factor structure of the MSQ v2.0 was evaluated and resulted in the removal of two items (MSQ v2.1) yielding a statistically improved factor structure compared to the 16-item MSQ v2.0 [11].

To facilitate patient use and data collection for the galcanezumab clinical studies, the MSQ v2.1 paper version was converted to the MSQ v2.1 ePRO for completion via the TrialSlate device, a tablet device with a touchscreen. Despite extensive evidence supporting the equivalence of electronic and paper PRO administration [12], instrument-specific evidence was needed to support the content validity and usability of the MSQ v2.1 ePRO in this context, as recommended per the Food and Drug Administration (FDA) PRO Guidance [13]. Therefore, a concept elicitation, cognitive debriefing, and usability study was undertaken to: 1) ascertain the migraine experience with a particular focus on the impact on roles and day-to-day functioning; 2) determine the comprehensiveness and comprehensibility of the MSQ v2.1 ePRO RFR domain items, as well as the appropriateness and understanding of the recall period, response options, and instructions; and 3) assess the usability of the MSQ v2.1 on an electronic tablet device (TrialSlate).

## Methods

### Instrument

**Fig. 1:**
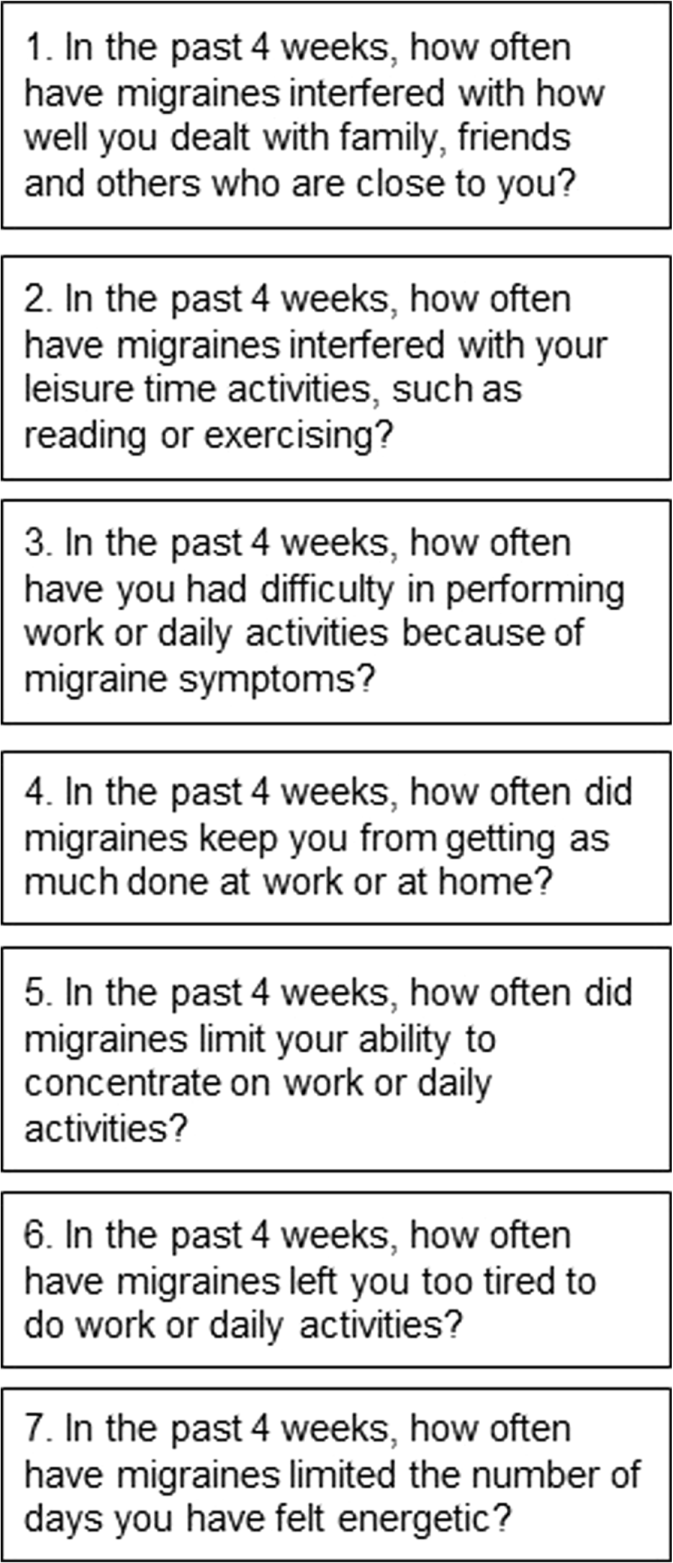
MSQ v2.1 ePRO RFR Domain Items

The MSQ v2.1 ePRO used in this study was an electronic version of the previously validated MSQ v2.1. The MSQ v2.1 is one of the most commonly used disease-specific tools for assessing the impact of migraine on quality of life (QOL) and functioning [14], and is a highly recommended data collection element by the National Institute of Neurological Disorders and Stroke [15]. The MSQ v2.1 ePRO contains 14 questions from three domains, with six response options that range from “None of the time” to “All of the time.” The MSQ v2.1 ePRO RFR domain contains seven items that measures the degree to which performance of day-to-day activities is restricted by migraine. Wording of the seven domain items is shown in Fig. 1. Each of the three MSQ domains are scored independently on a linearly-transformed scale from 0 to 100, with a higher score indicating a better health status.

### Participants

All interviews were conducted in a US English-speaking sample of people with chronic or episodic migraine. Participant inclusion criteria were similar to those used in the phase 3 galcanezumab clinical studies. Key criteria for the qualitative research included participants’ being ≥18 years old, having a diagnosis of migraine as defined by the International Headache Society (IHS) International Classification of Headache Disorders (ICHD)-3 beta guidelines, with history of migraine for at least one year, onset prior to age 50, and having a history of four to 14 migraine headache days and at least two migraine attacks per month on average within the past three months (episodic migraine), or having a history of ≥15 headache days per month for more than three months that has the features of migraine headache on at least eight days per month (chronic migraine).

All potentially eligible participants were screened for study eligibility using patient medical charts and were subsequently contacted by site staff using a standardized screening script, either over the telephone or at their regularly scheduled office visits. Eligible participants were invited to participate in a face-to-face interview. Purposive recruitment efforts were made so that at least one-third of the sample consisted of people with chronic migraine, and at least two participants were male. This study was approved by the Chesapeake Institutional Review Board; all participants provided written informed consent.

### Data collection and analysis

A semi-structured interview guide was used during the one-on-one interviews, encompassing methods appropriate for concept elicitation, cognitive debriefing, and usability testing. Each interview took between 90 and 120 min to complete. The objective of the concept elicitation portion of the interview was to understand participants’ episodic or chronic migraine experiences. This portion included open-ended questions about migraine-related symptoms, as well as the negative impacts or day-to-day effects associated with their migraine experience. Participants were also asked what they thought would be a meaningful change or improvement in their day-to-day effects or functioning (i.e., describe experiences or features that would be necessary to consider a treatment “beneficial” with regard to day-to-day effects).

Following the open-ended concept elicitation discussion, participants were asked to complete the MSQ v2.1 ePRO and then provide feedback on the overall comprehension, relevance, and content validity of the RFR domain, and answer a set of questions about device usability. The interviews were conducted in-person and were audio-recorded. Each participant was compensated $150 via ClinCard for their time immediately following the interview.

Qualitative data were analyzed using ATLAS.ti qualitative data analysis software version 7.5.18 [16]. ATLAS.ti software was designed for the qualitative analysis of textual, graphical, audio, and video data. ATLAS.ti allows the researcher to create and enter names of concepts, or “codes,” to be used for conceptualizing large amounts of qualitative data. The program allows the analyst to organize and relate these concepts to each other in order to evaluate the underlying structure of recurring themes or groupings of the qualitative data. The constant comparative method, an iterative coding approach moving between consecutive transcripts and new codes that emerge [17], was followed. The coders attached relevant “codes” to concepts mentioned within each transcript. Two transcripts were double-coded in full by study team members to assure a standardized coding approach. Coding outputs were generated from ATLAS.ti based on each utilized code. The utilized codes from the concept elicitation portion of the interview were entered into a saturation grid. Saturation is defined as the point at which no substantially new themes, descriptions of a concept, or terms are introduced as additional interviews are conducted [18, 19]. The number of participants needed to reach saturation is largely driven by the complexity of a concept and the diversity of the patient population (e.g., age, severity) who share the commonality of interest (e.g., disease, treatment, or other relevant health-related experience). While additional interviews can contribute to confidence in content validity, there is a point at which additional sampling offers no new information and serves no purpose; this is the point of saturation [20, 21].

## Results

Following the screening process across the three US clinical sites, a total of 15 potential participants were identified and approached for participation. Three individiuals were uninterested in participating, and one was deemed ineligible after confirmation of inclusion/exlusion criteria. A total of 11 participants took part in the qualitative study, including concept elicitation, cognitive debriefing, and usability testing described in the Methods section. Participants had a mean age of 34.8 (standard deviation [SD] = 7.7) years, and nine were female. The majority of the sample identified as white (10 of 11 participants) (Table 1). Four were participants with chronic migraine, and seven were participants with episodic migraine. Additional demographic and clinical characteristics, including comorbid conditions and migraine treatments, are found in Table 1.

**Table 1:** Qualitative research sample demographic and clinical characteristics

	Total sample (*N* = 11)	Episodic Migraine (*N* = 7)	Chronic Migraine (*N* = 4)
Demographics			
Age (years), Mean (SD) [min, max]	34.8 (7.7) [24, 53]	34.9 (9.2) [24, 53]	34.8 (5.3) [29, 41]
Female, n (%)	9 (81.8%)	6 (85.7%)	3 (75.0%)
Race, n (%)			
White	10 (90.9%)	6 (85.7%)	4 (100%)
Black or African American	1 (9.1%)	1 (14.3%)	
Ethnicity, n (%)			
Hispanic or Latino	1 (9.1%)	1 (14.3%)	
Not Hispanic or Latino	10 (90.9%)	6 (85.7%)	4 (100%)
Current Living Situation, n (%)			
Living alone	1 (9.1%)		1 (25.0%)
Living with a partner, spouse, family, or friends	10 (90.9%)	7 (100%)	3 (75.0%)
Education, n (%)			
Secondary/high school	1 (9.1%)	1 (14.3%)	
Some college	2 (18.2%)	1 (14.3%)	1 (25.5%)
College degree	2 (18.2%)	2 (28.6%)	
Postgraduate college	4 (36.4%)	2 (28.6%)	2 (50.0%)
Technical or vocational degree	2 (18.2%)	1 (14.3%)	1 (25.0%)
Employment Status, n (%)			
Employed full-time	7 (63.6%)	5 (71.4%)	2 (50.0%)
Employed part-time	2 (18.2%)	1 (14.3%)	1 (25.0%)
Homemaker	1 (9.1%)	1 (14.3%)	
Disability	1 (9.1%)		1 (25.0%)
Annual Household Income, n (%)			
$25,000 to $44,000	3 (27.3%)	2 (28.6%)	1 (25.0%)
$45,000 to $75,000	2 (18.2%)	2 (28.6%)	
More than $75,000	5 (45.5%)	2 (28.6%)	3 (75.0%)
Prefer not to say	1 (9.1%)	1 (14.3%)	
Clinical			
Episodic migraine, n (%)	7 (63.6%)		
Chronic migraine, n (%)	4 (36.4%)		
Time since diagnosis, n (%)			
Less than 1 year	0 (0%)	0 (0%)	0 (0%)
More than 1 year and up to 5 years	2 (18.2%)	1 (14.3%)	1 (25.0%)
More than 5 years and up to 10 years	3 (27.3%)	1 (14.3%)	2 (50.0%)
More than 10 years	6 (54.5%)	5 (71.4%)	1 (25.0%)
Comorbid conditions, n (%)^a^			
Anxiety	3 (27.3%)	2 (28.6%)	1 (25.0%)
Depressive disorder	4 (36.4%)	1 (14.3%)	3 (75.0%)
Obsessive compulsive disorder	1 (9.1%)	1 (14.3%)	
Ovarian cysts or other ovarian disorder	1 (9.1%)		1 (25.0%)
Asthma	1 (9.1%)		1 (25.0%)
No other health problems	2 (18.2%)	2 (28.6%)	

Abbreviations: *max* = maximum, *min* = minimum, *n* = number of patients within each specific category, *SD* = standard deviation

^a^Not mutually exclusive

### Concept elicitation

During the concept elicitation interviews, the migraine symptoms reported either spontaneously or using probing techniques included: head pain (front of head, back of head, sides of head), nausea, sensory sensitivity, neck pain, concentration or focus issues, and anxiety. For these migraine symptoms, saturation was reached within the first six interviews.

When queried about the effects that episodic or chronic migraine symptoms have on day-to-day activities, the impacts/impairments reported either spontaneously or via probing were: relationships/interactions with others, child care/parenting, leisure time activities, work/employment, work around the home, concentration or focus, fatigue/tiredness, energy level, psychological (i.e., anxiety, depression, or mood), and food and alcohol consumption (i.e., altered consumption or unable to consume) (Table 2). Results demonstrated that the content of the MSQ v2.1 ePRO RFR domain covered impacts/impairments that were important and relevant to patients with episodic or chronic migraine. Importantly, whether through spontaneous mention in the open-ended questioning or through probing, the concepts of the MSQ v2.1 ePRO RFR domain items were endorsed by all 11 participants; the only exception was item 5 (ability to concentrate), which was endorsed by 10 of the 11 participants. While participants gave many specific examples of how their day-to-day activities were affected by migraine attacks (Table 3), they also made general statements about the impact of migraine attacks: “I literally can’t do anything,” “I can’t function,” and “I’m incapable of doing anything pretty much”.

**Table 2:** Saturation grid of migraine effects on day-to-day activities

Concept	002–003	002-006^a^	002-005	002-002^a^	002-001	003-001	001-001	003–002	001–005	001-004^a^	001-006^a^
Psychological	S					S			S		S
Work or daily activities (work/employment, work around the home, driving)	S	S	S	S	S	S	S	S	S	S	S
Relationships/interactions with others (social, employment, parenting)	S	P	P	S	P	S	S	S	P	P	S
Food and alcohol consumption	S						S		S		
Leisure time activities (exercise, reading, socializing)	P	P	S	S	S	P	P	P	P	S	P
Concentration or focus	P	P	S	P	P	P	S	P		S	P
Fatigue/tiredness	P	P	S	S	P	P	S	S	P	S	P
Energy level	P	P	P	P	P	P	S	S	P	S	P

^a^Chronic migraine

**Table 3:** Qualitative support for MSQ v2.1 ePRO RFR domain concepts

MSQ v2.1 ePRO RFR domain Concepts	Example Quotes
Item 1: Interfere with family, friends, and others	*• At first some of my friends would think I didn’t want to really go out with them because, you know, it [cancelling] would happen repeatedly.* *• Definitely for me it affects how I feel my relationships with people are, you know.*
Item 2: Interfere with leisure time activities	*• Physical activity is something that I do for leisure. So, sometimes I can’t do that.* *• I--really I love to play tennis and golf and read, and so it [migraine attacks] affects all of those.*
Item 3: Difficulty performing work or daily activities	*• I’ve been at work before and had to either lay down and fight through them just for a little bit or, sometimes I’ve just had to leave work and go home.* *• I have to modify my lesson plan for my students, because I really can’t present a lot in front of them.* *• It’s just so hard for me to just function to drive—to drive a car, to do, you know, all these things that are normally required of people.* *• Work as I have been saying, I just force myself to power through it.*
Item 4: Keep you from getting as much done at work or at home	*• When I say I can’t function, I can’t cook. I can’t clean up anything. I can’t--just simple tasks that you would think, you know, talking on the phone. I can’t even look at my phone, because the light on the phone just sends me into a whirlwind of just like haze.* *• I couldn’t get things done… things for work.* *• I go to work even with or without a migraine attack. I’m sure I’m not able to work as efficiently.* *• I might have been able to get some stuff, but was I actually able to get all of it? And the general answer would be no. I wouldn’t be able to get everything I wanted done because I’m either moving slower, or I’m making mistakes.*
Item 5: Limit your ability to concentrate	*• I feel like you kind of lose a sense of, um, being able to focus, you know, because all you feel is my head hurts this bad. I don’t know what you’re saying to me right now or what, you know. Just the processing is slowed down for sure.* *• My ability to focus on what I’m trying to read and follow the words and everything has diminished a lot.*
Item 6: Left you too tired	*• You’re exhausted from how bad your head hurt, you know that whole time. So, I would definitely say, um, the majority of the fatigue is after the worst part of the pain has subsided.* *• Using an example of grading papers… I’m just too tired. I’m not going to do it, so.* *• I guess post-migraine attack residual headache, um, that’s like I’m so lethargic. I’m like a sloth.*
Item 7: Limited the number of days you felt energetic	*• It does affect the energy level. I—I mean, I, you know, most of the time I wake up in the morning even if it’s a really mild headache I feel like it’s like, okay. Here’s another headache, you know. I—I don’t have a—it definitely takes a lot of energy out of me.* *• When it’s [migraine attack] really bad, my energy is, you know, sinks pretty low.*

### Cognitive debriefing

Cognitive interviews confirmed that the MSQ v2.1 ePRO instructions were clear, and the items comprising the seven-item RFR domain captured the relevant daily functioning impacts experienced as a result of migraine. All participants reported that all seven items were meaningful and important to assess as an impact of migraine symptoms, and all participants desmonstrated understanding of each item, with the exception of one participant misunderstanding item 7 (described below).

With regard to item 1 (how often have your migraines interfered with how well you dealt with family, friends and others who are close to you), participants reported that migraine “affects how you deal with people, affects their relationships with others,” and it “does not just affect you.” Regarding item 2 (how often have your migraines interfered with your leisure time activities, such as reading or exercising) participants were asked examples of what they consider to be leisure time activities, and if they considered reading and exercise to be leisure time activities. A variety of leisure time activities were reported by participants, including reading and multiple types of exercise (for example, golf, yoga, basketball, running, walking). All but one participant reported that they considered reading and exercise to be leisure time activities. The single participant that responded in the negative stated that “it [exercise] is a chore.” Some specific examples provided by participants with regard to daily activities that they had difficulty performing (item 3: how often have you had difficulty in performing work or daily activities because of migraine symptoms) included child care, driving, shopping, cooking, and eating. Two participants reported that item 4 (how often did migraines keep you from getting as much done at work or at home) was similar or redundant to item 3. Some of the specific examples provided by participants in relation to not getting as much done at work or at home included paperwork, using the computer or cell phone, cleaning, laundry, and yard work. When probed, all but one participant found the concept of concentration (item 5: how often did migraines limit your ability to concentrate on work or daily activities) to be relevant to their migraine experience, although s/he did not explain why it was not relevant. Most participants (*n* = 7) spoke specifically about the difficulties with concentrating on reading or doing work on the computer. Some participants (*n* = 5) discussed how fatigue (item 6: how often have migraines left you too tired to do work or daily activities) was typically experienced after the migraine attack, and reported experiencing fatigue days after the migraine attack. Many participants (*n* = 6) reported they went to work despite having a migraine attack, but that they were too fatigued following work to perform other daily activities. Below is one specific example of how a migraine attack left a participant too tired to work:

“…Using an example of grading papers… I’m just too tired. I’m not going to do it, so.”

When debriefed on item 7 (how often have migraines limited the number of days you have felt energetic), one participant was confused by the word “limited.” The participant thought she had read the item wrong, but when asked to elaborate on her understanding, she did find the item to be clear and understandable. Two other participants reported that item 7 is similar to item 6; however, they both demonstrated understanding of the item and neither had alternative wording suggestions.

Ten of the 11 participants reported that they clearly understood the response options, and demonstrated that they were able to differentiate between the six options. One participant reported that there was no perceived difference between “some of the time” and “a good bit of the time.” This participant suggested that assigning numerical value or frequency to the response options would be “an easier way of doing it.” All participants confirmed that they could recall the four-week period; however, two participants did state that it could be difficult to recall accurately because their migraine attacks happen so frequently (“I get quite a few of them, so it’s a little difficult; it happened so frequently that I did my best to remember.”). To orient themselves to the four-week recall window and select a response option, some participants discussed using their work schedule or other memorable events. As an example, one participant stated: “I remembered that in those last four weeks it was my birthday. And then, I kind of worked backwards from all that.” As far as an alternative to the four-week recall period, two participants suggested using a two-week recall period. However, these suggestions were specific to only two items, with two participants making the suggestion for item 5 and item 6, respectively. Across the 11 participants and the seven RFR domain items, the four-week recall period was endorsed 97% (75/77) of the time.

### Usability testing

All participants reported they were familiar with technological devices such as computers, tablets, or smartphones, and used them on a regular basis. Most participants stated they were very comfortable using computers and smartphones, and three stated they had a basic or moderate level of comfort with them. Five of the 11 participants had previously completed a survey on a tablet device similar to the TrialSlate (which was used for the MSQ v2.1 ePRO administration), either in a medical setting or for human resources. Overall impressions participants had of the TrialSlate device were favorable. Almost all participants commented that it was “easy to use.” One participant thought the interface was “outdated” and the device was “physically clunky.” Some of the things participants liked about the TrialSlate included its size, that it was easy to read and to click on the screen, and it was not paper. No participants reported any difficulties with using the device (i.e., screen transition, answer option selection, viewing items and answer options) or in understanding the questions.

## Discussion

The 11 qualitative interviews demonstrated that participants with episodic and chronic migraine commonly experience symptoms of front, back, and side head pain; nausea; sensory sensitivity; neck pain; concentration or focus issues; and anxiety due to migraine. Participants also endorsed migraine attack impacts, including relationships/interactions with others, leisure time activities, work/employment, work around the home, child care/parenting, concentration or focus, fatigue/tiredness, and energy level, all of which are consistent with what has been documented in the literature [1, 22, 23]. These findings confirm that the MSQ v2.1 ePRO RFR domain concepts are important and relevant to participants with migraine, and comprehensive of the degree to which performance of day-to-day activities is limited by migraine.

Overall, participants provided positive feedback on the MSQ v2.1 ePRO instructions, items, and response options, and felt that they were comprehensive and relevant to their experience with migraine attacks. Two study participants found item 7 (how often have migraines limited the number of days you have felt energetic) to be redundant with item 6 (how often have migraines left you too tired to do work or daily activities), and one participant misunderstood the term “limited” in item 7, but demonstrated an understanding of the item during debriefing. As to the redundancy, the structural validity and contribution of each RFR item has been examined in multiple studies [10, 11, 24] and within the three galcanezumab clinical studies. Those results support the structural validity of the domain and relative contribution of each item to the domain’s internal consistency.

The four-week recall period was correctly understood and easy for participants to recall experiences of migraine attacks, as reported by all participants, and they were able to think of specific events (e.g., work schedule, vacation, birthday) when responding to each item. Four weeks is thus an appropriate period of assessment for migraine symptoms and impact. The four-week (or one-month) window is a clinically relevant timeframe that is the standard used in both research [25] and clinical practice (IHS 2013 [ICHD-3rd edition beta]) for the characterization of migraine pattern. Although there are no established absolute criteria for gauging response to migraine preventive therapy, an accepted definition for treatment response is ≥50% reduction in the number of migraine headache days in the four weeks prior to return for a follow-up visit [26]. Lipton and colleagues [27] published a review on the quality of health-related quality of life (HRQL) measures in headache and migraine. They found that many of the current migraine-specific instruments have a four-week recall interval, but some use a three-month interval, or a shorter two-week recall interval. These authors noted that while the two-week interval can provide more accurate recall for the period of measurement, it can be prone to temporal sampling error. Conversely, they stated that longer recall intervals are likely to capture a more representative sample of a patient’s migraine experience [27]. An important consideration and benefit of the four-week recall may be the ability to capture the interictal burden, or impairment between attacks [6].

There were benefits for the galcanezumab clinical trial program to using the MSQ v2.1 to assess the degree to which performance of day-to-day activities is restricted by migraine. As reported above, the publicly available MSQ v2.1 is one of the most commonly used disease-specific tools assessing the impact of migraine on HRQL [14] and is highly recommended by the National Institute of Neurological Disorders and Stroke [15]. Early development of the MSQ v1.0 was consistent with FDA PRO Guidance [13], and the subsequent work to refine the instrument resulted in a version with well-established measurement properties in both episodic and chronic migraine [11, 14].

The usability of the TrialSlate ePRO device used in the study was reported to be straightforward and easy to use. There are multiple advantages to using ePRO instead of “paper-and-pen” data collection methods. Evidence suggests that an ePRO platform results in more accurate and complete data, improved compliance, the ability to program skip patterns, avoidance of secondary data entry errors, less administrative burden, and high respondent acceptance [28]. In addition, there is a body of evidence that supports the equivalence of electronic and paper-and-pen data collection [12, 29]. Further, the FDA Guidance for Industry supports the use of ePRO data collection in clinical trials [30].

A limitation of this study was that only two of the respondents were male. While migraine is experienced three-to-one in females versus males [31], it may have been beneficial to recruit another male participant. That said, the saturation of concepts during the concept elicitation portion of the interview was reached by the sixth participant, and the cognitive debriefing and usability testing results were very clear and consistent across participants. In addition, published evidence demonstrates that men and women alike experience diminished quality of life, as well as a range of impairments pertaining to occupational, familial, and social functioning [32]. Other limitations with regard to generalizability of these findings are the limited racial and ethnic diversity among the study participants, and the fact that the vast majority were highly educated. Finally, given that this research was conducted in the United States only, future research among a more diverse patient population across multiple countries would be beneficial.

## Conclusions

This study adds to the existing evidence demonstrating content validity of the MSQ v2.1 ePRO RFR domain as a measure of impact of migraine on daily functioning in patients with episodic or chronic migraine. Further, this body of research provides evidence that the functional restrictions included in the MSQ v2.1 ePRO RFR domain are important to—and well understood by—people with migraine, and reflect the wide range of day-to-day impacts of migraine. The MSQ v2.1 ePRO RFR domain is content-valid and appropriate for inclusion in future studies designed to measure the restriction of episodic or chronic migraine performance of day-to-day activities.

## Data Availability

Please contact author for data requests.

## Abbreviations

ePRO: electronic patient-reported outcome
FDA: Food and Drug Administration
HIS: International Headache Society
HRQL: health-related quality of life
ICHD: International Classification of Headache Disorders
MSQ: Migraine-Specific Quality of Life Questionnaire
MSQ v2.1 ePRO RFR: Migraine-Specific Quality of Life Questionnaire version 2.1 electronic patient-reported outcome Role Function-Restrictive
PRO: Patient-reported outcome
QOL: Quality of life
SD: Standard deviation
